# Association between frailty status and delayed observation-room retention after painless gastrointestinal endoscopy in older patients

**DOI:** 10.3389/fmed.2026.1859679

**Published:** 2026-08-13

**Authors:** Dongxu Sun, Tingting Feng, Wudong Zhuang, Yazhao Sun, Fang Xing

**Affiliations:** 1 Department of Anesthesiology, Hebei Cangzhou Hospital of Integrated Traditional Chinese and Western Medicine, Cangzhou, Hebei, China; 2 Department of Cardiology, Cangzhou People's Hospital, Cangzhou, Hebei, China

**Keywords:** delayed observation-room retention, frailty status, older patients, painless gastrointestinal endoscopy, post-anesthesia recovery, risk stratification

## Abstract

**Background:**

Frailty reflects reduced physiological reserve and may be associated with slower post-anesthesia recovery in older patients. This study examined the association between frailty status and delayed observation-room retention after painless gastrointestinal endoscopy and explored its additional discriminative contribution beyond routinely collected clinical and peri-procedural variables.

**Methods:**

A total of 516 older patients undergoing painless gastrointestinal endoscopy were included, and all analyses reported in the abstract and main manuscript were based on this same cohort. Frailty status was assessed using the Fried frailty phenotype. The primary outcome was delayed observation-room retention, defined using the 75th percentile of observation-room retention time in the study population. Logistic regression models were used to evaluate the association between frailty status and delayed observation-room retention. Model 1 was unadjusted, Model 2 was the primary directed acyclic graph (DAG)-informed confounder-adjusted model, and Model 3 additionally included total propofol dose and intraoperative adverse events as peri-procedural factors. Receiver operating characteristic (ROC) curves, net reclassification improvement (NRI), integrated discrimination improvement (IDI), and decision curve analysis were used for an exploratory in-sample assessment of the additional discrimination provided by frailty status. Because the base model included information arising during the procedure, this analysis was not intended to establish a pre-procedural prediction tool. Sensitivity analyses examined observation-room retention time as a continuous outcome and used the 80th percentile threshold.

**Results:**

The 75th percentile of observation-room retention time was 37.00 min, and 151 patients were classified as having delayed observation-room retention. Compared with patients with normal retention, those with delayed retention had a higher proportion of frailty (72.8% vs. 33.2%, *p* < 0.001). Frailty was associated with delayed retention in the unadjusted model (OR 5.410, 95% CI: 3.556–8.231, *p* < 0.001) and in the primary DAG-adjusted model (OR 3.098, 95% CI: 1.490–6.442, *p* = 0.002). After additional adjustment for peri-procedural factors, the association was attenuated but remained statistically significant (OR 2.061, 95% CI: 1.160–3.662, *p* = 0.014). Frailty status alone had an AUC of 0.698 (95% CI: 0.655–0.742). In the exploratory peri-procedural model, adding frailty increased the AUC from 0.932 to 0.940 (absolute increase, 0.008; DeLong *p* = 0.044). The total NRI was 0.665 (95% CI: 0.397–0.998, *p* = 0.001), and the IDI was 0.021 (95% CI: 0.011–0.067, *p* = 0.001). Decision curve analysis showed higher apparent net benefit across selected threshold ranges.

**Conclusion:**

Frailty status was associated with delayed observation-room retention in older patients undergoing painless gastrointestinal endoscopy. Its additional discriminative contribution beyond a model containing clinical and peri-procedural variables was small in absolute AUC terms and remains exploratory. Frailty assessment may provide useful information for post-procedure observation planning, but prospective multicenter validation is required before clinical implementation.

## Introduction

The global population is projected to increase from approximately 7 billion to 9.3 billion by 2050 and 10.1 billion by 2,100, while the old-age dependency ratio is expected to double by 2050 and triple by 2,100 (1). This demographic transition has increased the healthcare burden associated with aging and highlights the need for peri-endoscopic management strategies tailored to older patients.

Gastrointestinal diseases are common among older patients, and gastrointestinal endoscopy is widely used for screening, diagnosis, and treatment. Painless gastrointestinal endoscopy has become increasingly common because it can reduce procedural discomfort and improve patient tolerance. However, older patients often have age-related physiological changes that may affect anesthetic response, procedural tolerance, and post-anesthesia recovery. For example, alterations in gastrointestinal pH, permeability, and transit time may influence drug absorption and examination quality (2). In addition, chronic diseases such as cardiovascular disease and diabetes are common in older patients and may increase the risks associated with painless gastrointestinal endoscopy, including cardiovascular complications (3). Age-related decline in physical function, including reduced muscle mass, decreased muscle strength, and impaired coordination, may also affect procedural cooperation and recovery after anesthesia. Previous evidence suggests that preoperative interventions such as strength training may improve muscle strength and health-related quality of life in older patients (4). Cognitive decline may further impair patients’ ability to understand peri-endoscopic instructions, requiring more careful communication and individualized management before and after the procedure.

Frailty is a common geriatric syndrome characterized by reduced physiological reserve and impaired homeostatic regulation (5). It is associated with decreased functional capacity, increased vulnerability to stressors, and adverse clinical outcomes, including delayed recovery, complications, and mortality (6–9). With population aging, the number of frail older patients continues to increase, making frailty an important issue in peri-endoscopic risk assessment (10). Although the relationship between frailty and postoperative outcomes has been extensively investigated in surgical populations, evidence remains limited regarding the role of frailty status in post-anesthesia recovery after painless gastrointestinal endoscopy. Observation-room retention time reflects the recovery process after painless gastrointestinal endoscopy and may be influenced by patient vulnerability, anesthetic exposure, procedural characteristics, and peri-endoscopic adverse events. Delayed observation-room retention may increase monitoring demands and affect post-procedure workflow. However, the association between frailty and delayed observation-room retention after accounting for baseline characteristics and peri-procedural factors, and the extent to which frailty adds discrimination to such models, remain insufficiently understood.

Therefore, this study aimed to evaluate the association between frailty status and delayed observation-room retention in older patients undergoing painless gastrointestinal endoscopy. As a secondary, exploratory objective, we assessed whether adding frailty status to a model containing clinical and peri-procedural covariates improved discrimination for delayed observation-room retention. This analysis was intended to quantify the incremental information provided by frailty within the study cohort, rather than to develop or validate a pre-procedural risk prediction tool.

## Methods

### Study population

This study included older patients who underwent painless gastrointestinal endoscopy at Hebei Cangzhou Hospital of Integrated Traditional Chinese and Western Medicine between January 2025 and March 2026. Patients were eligible if they were aged ≥60 years and underwent gastroscopy, colonoscopy, or combined gastroscopy and colonoscopy under intravenous anesthesia. Patients were excluded if they had an American Society of Anesthesiologists (ASA) physical status of ≥IV, underwent an emergency or non-standard endoscopic procedure, such as polypectomy or foreign-body removal, had severe cognitive impairment or physical disability that precluded frailty assessment, developed major post-procedure complications, had incomplete clinical data, or had not provided broad written informed consent for the use of their clinical data for research purposes. After application of the eligibility criteria, 516 patients were included in the final analytic cohort. The study protocol was approved by the Hebei Cangzhou Hospital of Integrated Traditional Chinese and Western Medicine (approval number: CZX2026-KY060-02). The study was conducted in accordance with the Declaration of Helsinki. All participants provided broad written informed consent authorizing the use of their clinical data for research purposes.

### Painless gastrointestinal endoscopy and anesthesia management

All patients underwent standard pre-procedural preparation before painless gastrointestinal endoscopy. Intravenous access was established before the procedure, and routine monitoring was performed throughout anesthesia, including blood pressure, heart rate, peripheral oxygen saturation, and respiratory status. Painless gastrointestinal endoscopy was performed under intravenous anesthesia using propofol combined with remifentanil. Anesthesia induction and maintenance were performed according to clinical practice, and additional propofol was administered when necessary according to the depth of anesthesia and procedural requirements. During the procedure, hypotension, hypoxemia, bradycardia, aspiration, and other intraoperative adverse events were recorded and managed promptly according to standard clinical protocols. After the procedure, patients were transferred to the observation room for post-anesthesia monitoring until they met the discharge criteria. The discharge criteria included recovery of consciousness, stable vital signs, adequate oxygen saturation, and absence of clinically significant post-procedure discomfort or adverse reactions requiring further observation.

### Frailty assessment and covariates

Frailty status was assessed using the Fried frailty phenotype (11). The assessment included five components: unintentional weight loss, self-reported exhaustion, low physical activity, weak grip strength, and slow walking speed. Patients meeting ≥3 criteria were classified as frail, whereas those meeting fewer than 3 criteria were classified as non-frail. Frailty status was used as the primary exposure variable in this study.

Demographic and clinical variables included age, sex, body mass index (BMI), American Society of Anesthesiologists (ASA) physical status, smoking, alcohol consumption, motion sickness, sleep disorder, multimorbidity, baseline peripheral oxygen saturation (SpO_2_), baseline systolic blood pressure (SBP), and baseline heart rate (HR). Multimorbidity was defined as the presence of ≥2 chronic diseases.

Procedural and anesthesia-related variables included examination type, fasting duration, previous painless endoscopy, procedure time, total propofol dose, weight-adjusted propofol dose, total remifentanil dose, weight-adjusted remifentanil dose, and intraoperative adverse events.

### Outcome definition

The primary outcome was delayed observation-room retention. Observation-room retention time was defined as the time from entry into the observation room after painless gastrointestinal endoscopy to fulfillment of discharge criteria. The threshold for delayed observation-room retention was determined according to the 75th percentile of observation-room retention time in the study population. Because no universally accepted cutoff is available for this setting, the 75th percentile was used as a study-specific, data-driven operational definition.

### Statistical analysis

**Figure 1:**
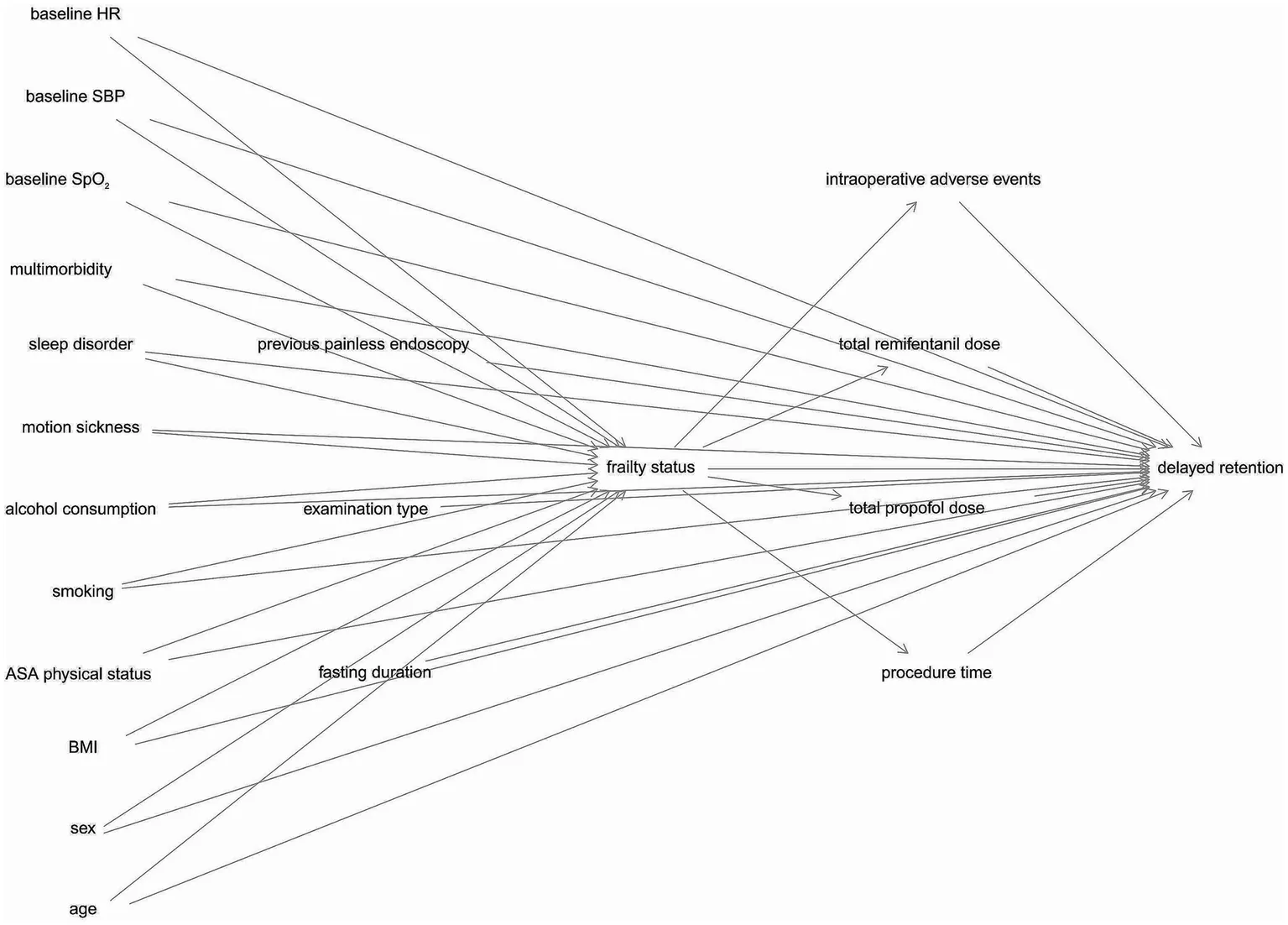
DAG for the association between frailty status and delayed observation-room retention. The DAG illustrates the assumed relationships among frailty status, delayed observation-room retention, baseline clinical characteristics, procedural factors, and anesthesia-related variables. Frailty status was the exposure of interest, and delayed observation-room retention was the outcome. The DAG-informed adjustment variables included age, sex, BMI, ASA physical status, alcohol consumption, smoking, motion sickness, sleep disorder, multimorbidity, baseline SpO_2_, baseline SBP, and baseline HR. These variables were included in the DAG-adjusted model. ASA, American Society of Anesthesiologists; BMI, body mass index; DAG, directed acyclic graph; HR, heart rate; SBP, systolic blood pressure; SpO₂, peripheral oxygen saturation.

All statistical analyses in this study were performed using R software (version 4.4.2). Continuous variables were presented as mean ± standard deviation (SD) or median with interquartile range (IQR), as appropriate, and categorical variables were presented as frequencies and percentages. Between-group comparisons were performed using Student’s t test or the Mann–Whitney U test for continuous variables and the chi-square test or Fisher’s exact test for categorical variables, as appropriate. All statistical tests were two-sided, and *p* values <0.05 were considered statistically significant. A directed acyclic graph (DAG) was used to identify covariates for the main adjusted model. The DAG-informed adjustment variables included age, sex, BMI, ASA physical status, alcohol consumption, smoking, motion sickness, sleep disorder, multimorbidity, baseline SpO_2_, baseline SBP, and baseline HR (Figure 1).

Logistic regression models were used to evaluate the association between frailty status and delayed observation-room retention. Model 1 was unadjusted. Model 2, designated as the primary confounder-adjusted model, included the DAG-informed covariates of age, sex, BMI, ASA physical status, alcohol consumption, smoking, motion sickness, sleep disorder, multimorbidity, baseline SpO2, baseline SBP, and baseline HR. Model 3 additionally included total propofol dose and intraoperative adverse events to examine attenuation of the association after accounting for peri-procedural factors. Because these variables arise during the procedure and may lie partly on the pathway between frailty and recovery, Model 3 was interpreted as an exploratory peri-procedural model rather than as an estimate of the total causal effect. Procedure time was not included in any multivariable regression model. Odds ratios (ORs) and 95% confidence intervals (CIs) were calculated. Multicollinearity was assessed using variance inflation factors, with values <5 considered acceptable.

The discriminatory ability of frailty status alone for delayed observation-room retention was evaluated using receiver operating characteristic (ROC) analysis and the area under the curve (AUC). An exploratory incremental discrimination analysis compared a base model containing the Model 3 covariates, except frailty status, with an extended model that additionally included frailty status. The base model therefore included total propofol dose and intraoperative adverse events, but it did not include procedure time or any component of the outcome. Because the two peri-procedural variables become available only during the procedure, this analysis characterized discrimination after peri-procedural information was available and was not intended as a pre-procedural prediction model. Model discrimination was assessed using ROC curves and AUCs, with AUCs compared using the DeLong test. Risk reclassification was evaluated using NRI and IDI, and apparent net benefit was examined using decision curve analysis. These indices were estimated in the same cohort used to fit the models and were therefore interpreted as exploratory. Two sensitivity analyses were performed. First, observation-room retention time was analyzed as a continuous outcome after natural logarithmic transformation using a Model 3-adjusted linear regression model. Second, delayed observation-room retention was redefined using the 80th percentile of observation-room retention time, and the association with frailty was re-examined using a Model 3-adjusted logistic regression model.

## Results

### Participant characteristics

A total of 516 older patients undergoing painless gastrointestinal endoscopy were included in the final analysis, and all results reported below refer to this same analytic cohort. The 75th percentile of observation-room retention time was 37.00 min; therefore, delayed observation-room retention was defined as an observation-room retention time ≥37.00 min. According to this definition, 365 patients were classified as having normal retention and 151 patients were classified as having delayed observation-room retention. The baseline characteristics of the study population are shown in Table 1. Compared with patients with normal retention, those with delayed observation-room retention were older, had a higher proportion of ASA III physical status, and were more likely to have motion sickness, sleep disorder, multimorbidity, and frailty. The proportion of frail patients was 72.8% in the delayed retention group, compared with 33.2% in the normal retention group (*p* < 0.001).

**Table 1:** Baseline characteristics of the patients studied.

Variable	Level	Overall (*n* = 516)	Normal retention (*n* = 365)	Delayed retention (*n* = 151)	*p* value
Age, years, mean ± SD		67.73 ± 5.14	66.88 ± 4.86	69.79 ± 5.23	<0.001
Sex, *n* (%)	Female	172 (33.3)	127 (34.8)	45 (29.8)	0.321
	Male	344 (66.7)	238 (65.2)	106 (70.2)	
BMI, kg/m^2^, mean ± SD		24.44 ± 1.68	24.40 ± 1.68	24.54 ± 1.66	0.399
ASA physical status, *n* (%)	ASA I	107 (20.7)	78 (21.4)	29 (19.2)	0.011
	ASA II	268 (51.9)	201 (55.1)	67 (44.4)	
	ASA III	141 (27.3)	86 (23.6)	55 (36.4)	
Smoking, *n* (%)	No	387 (75.0)	267 (73.2)	120 (79.5)	0.163
	Yes	129 (25.0)	98 (26.8)	31 (20.5)	
Alcohol consumption, *n* (%)	No	456 (88.4)	325 (89.0)	131 (86.8)	0.558
	Yes	60 (11.6)	40 (11.0)	20 (13.2)	
Motion sickness, *n* (%)	No	472 (91.5)	349 (95.6)	123 (81.5)	<0.001
	Yes	44 (8.5)	16 (4.4)	28 (18.5)	
Sleep disorder, *n* (%)	No	423 (82.0)	320 (87.7)	103 (68.2)	<0.001
	Yes	93 (18.0)	45 (12.3)	48 (31.8)	
Multimorbidity, *n* (%)	No	281 (54.5)	243 (66.6)	38 (25.2)	<0.001
	Yes	235 (45.5)	122 (33.4)	113 (74.8)	
Frailty status, *n* (%)	Non-frail	285 (55.2)	244 (66.8)	41 (27.2)	<0.001
	Frail	231 (44.8)	121 (33.2)	110 (72.8)	
Baseline SpO_2_, %, mean ± SD		96.43 ± 1.56	96.87 ± 1.36	95.35 ± 1.50	<0.001
Baseline SBP, mmHg, mean ± SD		123.28 ± 10.06	122.17 ± 9.93	125.94 ± 9.92	<0.001
Baseline HR, bpm, mean ± SD		75.54 ± 7.07	74.32 ± 7.01	78.49 ± 6.32	<0.001
Fasting duration, h, median (IQR)		8.00 (8.00, 10.00)	8.00 (8.00, 10.00)	8.00 (8.00, 10.00)	0.683
Examination type, *n* (%)	Gastroscopy	292 (56.6)	247 (67.7)	45 (29.8)	<0.001
	Colonoscopy	125 (24.2)	81 (22.2)	44 (29.1)	
	Gastroscopy + colonoscopy	99 (19.2)	37 (10.1)	62 (41.1)	
Previous painless endoscopy, *n* (%)	No	479 (92.8)	343 (94.0)	136 (90.1)	0.168
	Yes	37 (7.2)	22 (6.0)	15 (9.9)	
Procedure time, min, median (IQR)		7.00 (6.00, 13.00)	7.00 (6.00, 11.00)	13.00 (7.00, 20.00)	<0.001
Total propofol dose, mg, median (IQR)		48.75 (37.60, 66.25)	44.40 (35.20, 56.60)	68.20 (51.70, 90.95)	<0.001
Propofol dose, mg/kg, median (IQR)		0.72 (0.56, 0.97)	0.70 (0.54, 0.84)	0.99 (0.72, 1.36)	<0.001
Total remifentanil dose, μg, median (IQR)		27.80 (24.50, 32.15)	26.50 (23.60, 30.20)	31.30 (27.75, 36.40)	<0.001
Remifentanil dose, μg/kg, median (IQR)		0.41 (0.38, 0.47)	0.40 (0.37, 0.44)	0.47 (0.41, 0.55)	<0.001
Intraoperative adverse events, *n* (%)	No	391 (75.8)	324 (88.8)	67 (44.4)	<0.001
	Yes	125 (24.2)	41 (11.2)	84 (55.6)	

ASA, American Society of Anesthesiologists; BMI, body mass index; HR, heart rate; IQR, interquartile range; SBP, systolic blood pressure; SD, standard deviation; SpO_2_, peripheral oxygen saturation.

In addition, patients with delayed observation-room retention had lower baseline SpO_2_, higher baseline SBP, and higher baseline HR. Regarding procedural and anesthesia-related characteristics, patients with delayed observation-room retention had a higher proportion of combined gastroscopy and colonoscopy, longer procedure time, higher total propofol dose, higher weight-adjusted propofol dose, higher total remifentanil dose, and higher weight-adjusted remifentanil dose. Intraoperative adverse events were also more common in the delayed retention group. No significant differences were observed between the two groups in sex, BMI, smoking, alcohol consumption, fasting duration, or previous painless endoscopy.

### Association between frailty and delayed observation-room retention

Three logistic regression models were used to examine the association between frailty status and delayed observation-room retention (Table 2). In the unadjusted model, frail patients had higher odds of delayed retention than non-frail patients (OR 5.410, 95% CI: 3.556–8.231, *p* < 0.001). In the primary DAG-adjusted model, the association remained statistically significant (OR 3.098, 95% CI: 1.490–6.442, *p* = 0.002). After total propofol dose and intraoperative adverse events were added, the estimate was attenuated but remained statistically significant (OR 2.061, 95% CI: 1.160–3.662, *p* = 0.014). The differing effect estimates therefore reflect progressive adjustment across the three specified models rather than different analytic samples. Because the additional Model 3 variables may be downstream of frailty, this model was interpreted as an exploratory peri-procedural attenuation analysis. Variance inflation factors for all Model 3 variables were <5, indicating no apparent multicollinearity. Frailty status alone showed fair discrimination for delayed retention, with an AUC of 0.698 (95% CI: 0.655–0.742).

**Table 2:** Association between frailty status and delayed observation-room retention.

Model	OR (95% CI)	*p* value
Model 1: Crude model	5.410 (3.556–8.231)	< 0.001
Model 2: DAG-adjusted model	3.098 (1.490–6.442)	0.002
Model 3: Exploratory peri-procedural model	2.061 (1.160–3.662)	0.014

Model 1 was unadjusted. Model 2 was the primary confounder-adjusted model and included covariates identified using a DAG: age, sex, BMI, ASA physical status, alcohol consumption, smoking, motion sickness, sleep disorder, multimorbidity, baseline SpO₂, baseline SBP, and baseline HR. Model 3 additionally included total propofol dose and intraoperative adverse events and was interpreted as an exploratory peri-procedural model because these factors arise during the procedure and may partly mediate the association between frailty and recovery. Procedure time was not included in any model.

ASA, American Society of Anesthesiologists; BMI, body mass index; DAG, directed acyclic graph; OR, odds ratio; CI, confidence interval; SBP, systolic blood pressure; SpO_2_, peripheral oxygen saturation; HR, heart rate.

### Exploratory incremental discrimination analysis

We further examined whether adding frailty status to the clinical and peri-procedural covariate model improved discrimination for delayed observation-room retention. The base model included the covariates in Model 3, excluding frailty status, and the extended model additionally included frailty status. Total propofol dose and intraoperative adverse events were included in both models, whereas procedure time was not included.

**Figure 2:**
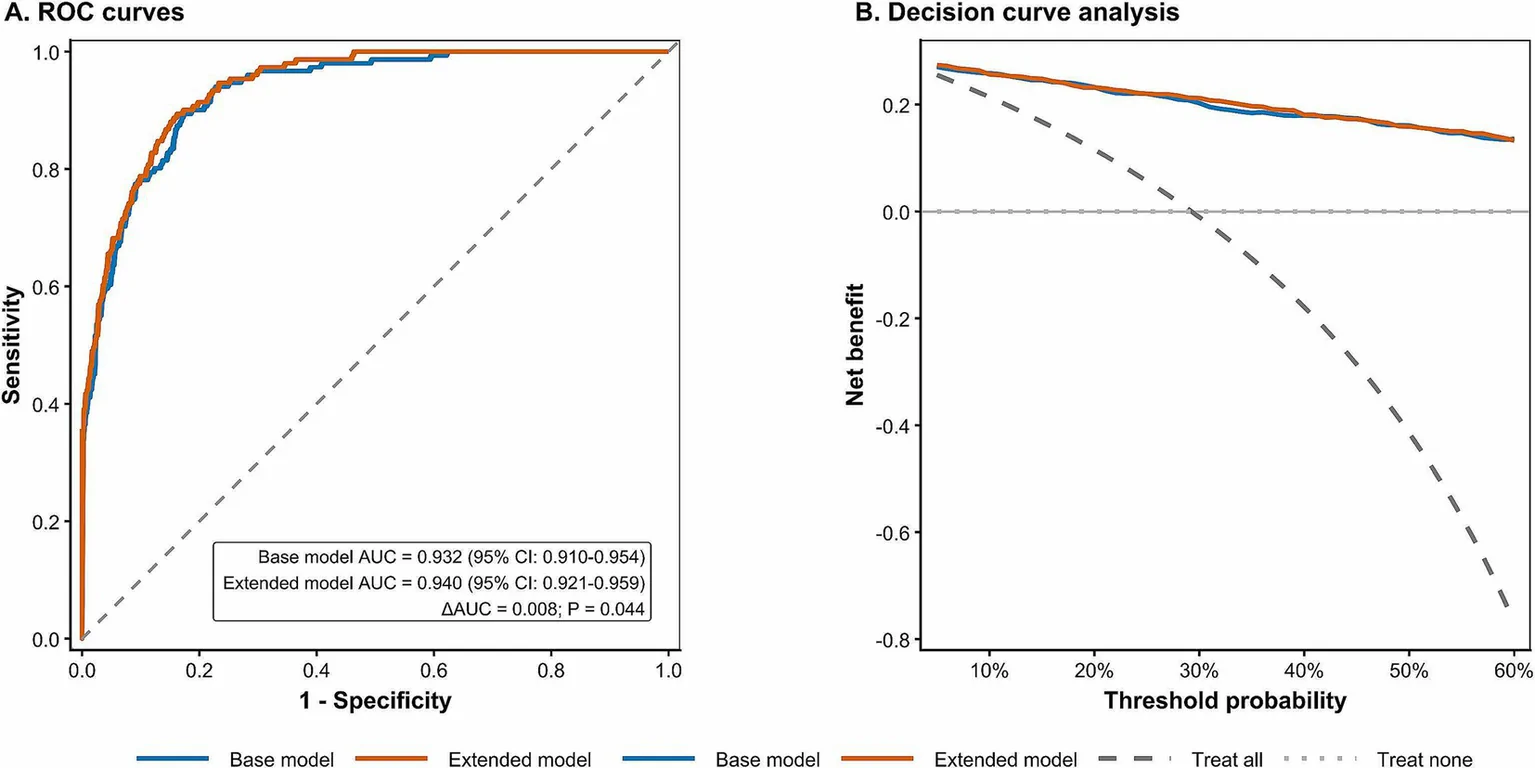
Exploratory in-sample discrimination and decision curve analyses after adding frailty status. **(A)** ROC curves comparing the base and extended models. **(B)** Decision curve analysis comparing the apparent net benefit of the base model, extended model, treat-all strategy, and treat-none strategy. The base model contained the Model 3 covariates except frailty status and therefore included total propofol dose and intraoperative adverse events, but not procedure time. The extended model additionally included frailty status. These analyses were conducted in the same cohort used to fit the models and were not intended to represent a pre-procedural prediction tool. ROC, receiver operating characteristic curve; AUC, area under the curve; CI, confidence interval.

The AUC increased from 0.932 (95% CI: 0.910–0.954) for the base model to 0.940 (95% CI: 0.921–0.959) for the extended model, corresponding to an absolute increase of 0.008 (DeLong test, *p* = 0.044). After frailty status was added, the event NRI was 0.311 (95% CI: 0.137–0.509, *p* = 0.002), the non-event NRI was 0.353 (95% CI: 0.165–0.557, *p* = 0.002), and the total NRI was 0.665 (95% CI: 0.397–0.998, *p* = 0.001). The IDI was 0.021 (95% CI: 0.011–0.067, *p* = 0.001) (Figure 2A; Table 3). Decision curve analysis showed that the extended model had a higher net benefit than the base model at threshold probability ranges of 5–9%, 12–16%, 21–24%, 26–41%, 47–48%, and 53–59%. The extended model also showed a higher net benefit than the treat-all and treat-none strategies across threshold probabilities of 5–60% (Figure 2B).

**Table 3:** Exploratory in-sample reclassification measures after adding frailty status to the base model.

Measure	Effect estimate	95% CI	*p* value
Event NRI	0.311	0.137–0.509	0.002
Non-event NRI	0.353	0.165–0.557	0.002
Total NRI	0.665	0.397–0.998	0.001
IDI	0.021	0.011–0.067	0.001

The base model contained the clinical and peri-procedural covariates included in Model 3, excluding frailty status; the extended model additionally included frailty status. Because the base model included total propofol dose and intraoperative adverse events, the results describe in-sample discrimination after peri-procedural information was available and should not be interpreted as pre-procedural predictive performance.

CI, confidence interval; IDI, integrated discrimination improvement; NRI, net reclassification improvement.

### Sensitivity analysis

To further assess the robustness of the association findings, two sensitivity analyses were performed. First, observation-room retention time was analyzed as a continuous outcome after natural logarithmic transformation and entered into a linear regression model. After adjustment according to the exploratory Model 3 specification, frail patients had a 13.6% longer observation-room retention time than non-frail patients (95% CI: 10.2–17.1%, *p* < 0.001).

Second, delayed observation-room retention was redefined using the 80th percentile of observation-room retention time as a more stringent data-driven threshold. The corresponding threshold was 39.00 min. Under this definition, 106 patients were classified as having delayed observation-room retention, accounting for 20.5% of the study population. In the exploratory Model 3-adjusted logistic regression analysis, frailty status remained associated with delayed observation-room retention (OR 1.904, 95% CI: 1.003–3.615, *p* = 0.049). These results were consistent with the primary association analysis.

## Discussion

In this single-center observational study, frailty status was associated with delayed observation-room retention after painless gastrointestinal endoscopy in older patients. The association was present in the primary DAG-adjusted model (OR 3.098) and was attenuated but remained statistically significant after additional adjustment for total propofol dose and intraoperative adverse events (OR 2.061). Frailty alone showed fair discrimination. Adding frailty to the peri-procedural base model produced a small absolute increase in AUC, while NRI, IDI, and decision curve analysis showed statistically detectable in-sample differences. These secondary findings should be interpreted as exploratory rather than as evidence of a validated clinical prediction model. The association findings were supported by analyses of retention time as a continuous outcome and by use of the 80th percentile threshold.

Frailty is a complex geriatric syndrome characterized by reduced physiological reserve, impaired homeostatic regulation, and increased vulnerability to stressors (12). Its pathophysiological features are closely related to oxidative stress, chronic inflammation, metabolic dysregulation, and multi-system functional decline (13). With aging, older patients often experience endocrine alterations, increased oxidative stress, heightened inflammatory responses, multimorbidity, and malnutrition. These factors may collectively impair organ reserve and reduce the ability to maintain physiological stability under procedural or anesthetic stress (14). Although painless gastrointestinal endoscopy is generally considered minimally invasive, it still involves fasting, sedative exposure, procedural stimulation, and post-anesthesia monitoring. Frail patients may therefore require longer observation before achieving adequate recovery and meeting discharge criteria.

In the context of painless gastrointestinal endoscopy, delayed observation-room retention may reflect slower recovery of consciousness, delayed stabilization of vital signs, residual sedative effects, or reduced tolerance to transient procedure-related physiological changes. Previous evidence has suggested that a higher frailty burden is associated with worse short-term outcomes after acute medical or surgical events (15), and that frailty may influence recovery trajectories after acute care (16). In the present study, the association between frailty status and delayed observation-room retention persisted after adjustment for total propofol dose and intraoperative adverse events, suggesting that frailty status may capture patient vulnerability beyond anesthesia exposure and immediate intraoperative complications.

Several mechanisms may explain the association between frailty status and delayed observation-room retention. First, frail patients have reduced cardiovascular, respiratory, and metabolic reserves, which may impair their ability to respond to anesthesia-related and procedure-related stress. Even mild hemodynamic fluctuation or oxygen desaturation may delay recovery and prolong monitoring time in the observation room. Second, frailty is closely related to immune dysfunction and chronic low-grade inflammation (17, 18). These conditions may reduce tolerance to procedural stress and delay restoration of physiological stability after anesthesia. Third, frailty often coexists with multimorbidity, which may further increase vulnerability and affect drug metabolism, recovery capacity, and post-procedure monitoring needs (19). Finally, frailty is associated with neuromuscular dysfunction and cognitive impairment, which may affect mobility, responsiveness, and the ability to follow post-procedure instructions, thereby contributing to delayed observation-room retention (20).

From a clinical perspective, the observed association suggests that frailty assessment may help identify older patients who warrant more deliberate recovery observation after painless gastrointestinal endoscopy. Observation-room retention time reflects both post-anesthesia recovery and the monitoring demands placed on an endoscopy unit. Nevertheless, this study does not establish that frailty-guided changes in anesthesia or discharge management improve patient outcomes or workflow efficiency. Such management strategies require prospective evaluation before routine implementation. The high AUC of the base model also requires careful interpretation. That model included total propofol dose and intraoperative adverse events, which occur during the procedure and are temporally close to recovery; these variables may function as markers of the developing recovery course and may partly mediate the effect of frailty. Their inclusion probably contributed to the strong base-model discrimination and prevents the analysis from being interpreted as a pre-procedural risk model. Procedure time was not included. Although adding frailty yielded statistically significant changes in AUC, NRI, and IDI, the absolute AUC gain was only 0.008, and the clinical importance of the reclassification measures is uncertain. The decision curve findings were likewise derived in the same cohort. Taken together, the incremental analyses are best viewed as hypothesis-generating and require independent validation.

## Limitations

This study has several limitations. First, its observational design does not permit causal inference, and residual confounding may remain despite DAG-informed adjustment. In addition, total propofol dose and intraoperative adverse events may lie partly on the pathway between frailty and recovery; accordingly, Model 3 should not be interpreted as estimating the total causal effect of frailty. Second, this was a single-center study, which limits generalizability. Third, delayed observation-room retention was defined using a study-specific percentile threshold rather than an externally established clinical cutoff. Fourth, the discrimination, reclassification, and decision curve analyses were developed and evaluated in the same cohort without external validation, so their apparent performance may be optimistic. The high AUC of the base model likely reflects, at least in part, inclusion of total propofol dose and intraoperative adverse events, which are temporally close to the recovery outcome. The small AUC increment after adding frailty and the clinical importance of the NRI and IDI therefore require cautious interpretation. Fifth, objective depth-of-anesthesia monitoring, such as Bispectral Index or entropy monitoring, was not used; sedation depth and supplemental propofol administration were guided by routine clinical assessment, which may have introduced variability. Sixth, frailty was assessed only with the Fried phenotype, and the findings may not generalize to other frailty instruments. Prospective multicenter studies with independent validation are needed to confirm the association and determine whether frailty-informed management improves recovery or resource use.

## Conclusion

Frailty status was associated with delayed observation-room retention in older patients undergoing painless gastrointestinal endoscopy. The association persisted after adjustment for baseline characteristics and was attenuated after additional peri-procedural adjustment. The added discrimination provided by frailty was small in absolute AUC terms, and the reclassification and decision curve findings remain exploratory. Frailty assessment may help inform future approaches to post-procedure observation, but prospective multicenter validation is needed before frailty-based management can be recommended.

## Data Availability

The original contributions presented in the study are included in the article, further inquiries can be directed to the corresponding author.
